# Single-cell mapping of leukocyte immunoglobulin-like receptors in kidney transplant rejection

**DOI:** 10.3389/frtra.2022.952785

**Published:** 2022-08-11

**Authors:** Baptiste Lamarthée, Coraline Genet, Florine Cattin, Richard Danger, Magali Giral, Sophie Brouard, Elisabet Van Loon, Jasper Callemeyn, Maarten Naesens, Dany Anglicheau, Bernard Bonnotte, Mathieu Legendre, Jean-Michel Rebibou, Claire Tinel

**Affiliations:** ^1^Université Bourgogne Franche-Comté, Etablissement Français du Sang Bourgogne Franche-Comté, Inserm UMR1098, RIGHT Interactions Greffon-Hôte-Tumeur/Ingénierie Cellulaire et Génique, Besançon, France; ^2^Nantes Université, CHU Nantes, Inserm UMR 1064, ITUN Center for Research in Transplantation and Translational Immunology (CR2TI), Nantes, France; ^3^Nephrology and Renal Transplantation Research Group, Department of Microbiology, Immunology and Transplantation, KU Leuven, Leuven, Belgium; ^4^Université de Paris Cité, Necker-Enfants Malades Institute, Inserm U1151, Paris, France; ^5^Department of Internal Medicine, Dijon University Hospital, Dijon, France; ^6^Department of Nephrology and Kidney Transplantation, Dijon University Hospital, Dijon, France

**Keywords:** kidney transplantation, single-cell RNA sequencing, LILR, monocytes, allorecognition

## Abstract

Leukocyte immunoglobulin-like receptors (LILRs) are a family of inhibitory or stimulatory receptors expressed by immune cell types belonging to both myeloid and lymphoid lineage. Several members of the LILR family recognize major histocompatibility complex class I and thus play important roles in a range of clinical situations including pregnancy. Moreover, paired immunoglobulin-like receptors (PIRs), the murine orthologs of LILRs, are implicated in experimental transplant allorecognition by monocytes and contribute to the induction of donor-specific monocyte-memory. After non-self recognition, activating PIRs are transiently overexpressed at the surface of monocytes and participate in donor-specific monocyte recruitment, leading to graft rejection *in vivo*. In the present study, we mapped LILR expression and also their respective reported ligands at single cell level in the renal allograft and circulating cells in the context of kidney transplant rejection. Recipient-derived monocytes were shown to infiltrate the donor tissue and to differentiate into macrophages. We thus also investigate LILR expression during *in vitro* monocyte-to-macrophage differentiation in order to characterize the myeloid population that directly contribute to allorecognition. Altogether our results emphasize non-classical monocytes and CD68+ M1 macrophages as key players in LILRs-ligand interaction in kidney transplantation.

## Introduction

Immune-mediated allograft injury remains a major hurdle to the long-term acceptance of solid-organ transplants and, after kidney transplantation, alloimmune injuries remain significant determinants of allograft failure (1, 2). Our view of the alloimmune response is usually dichotomized into T-cell-mediated rejection and antibody-mediated rejection (ABMR). The current ABMR classification requires the association of serologic evidence of circulating donor-specific antibodies (DSAs) and histologic lesions suggestive of tissue injury, particularly including evidence of recent antibody interaction with the endothelium (3). So far, antibody-targeting therapies fail to reverse ABMR. In order to find new therapeutic targets, better insight is needed into the biological mechanisms underlying ABMR processes. Recent reports suggest alternative mechanisms, designating monocytes/macrophages as non-humoral immune mediators of histological lesions in ABMR (4–6). Notably, in murine models of experimental transplant rejection, monocytes/macrophages were able to recognize the allograft through paired immunoglobulin-like receptors (PIRs). Indeed, after non-self recognition, activating PIRs are transiently overexpressed at the surface of murine monocytes and contribute to donor-specific monocyte recruitment, leading to graft rejection (4). The human orthologs of PIRs are leukocyte immunoglobulin-like receptors (LILRs). LILRs are a family of inhibitory or stimulatory receptors expressed by immune cell types of both myeloid and lymphoid lineage. Several members of the LILR family recognize major histocompatibility complex (MHC) class I peptides and thus play an important role in a range of clinical situations including pregnancy (7). In the present study, we used available online data to map the expression of LILRs using single-cell RNA sequencing for peripheral blood mononuclear cells (PBMC) and kidney allograft-derived cells. We also investigated the expression of the LILR members in monocytes and *in vitro* monocyte-derived populations.

## Materials and methods

### Single-cell RNA-sequencing data analysis

For PBMC, original data were generated as recently described (8). Briefly, blood samples were collected at time of indication biopsy or protocol biopsy at Nantes' hospital, France. They were isolated using density gradient and frozen in their respective serum complemented with 10%DMSO for storage in liquid nitrogen and shipment. Upon arrival in Leuven, Belgium, they were then thawed, centrifuged at 200 g for 5 min at 4°C before resuspension in PBS containing 0.04% UltraPure BSA (AM2616, ThermoFisher Scientific) and strained through a 40 μm cell strainer to further remove cell clumps and large fragments. Libraries were generated using the Chromium Single Cell 5′ library and Gel Bead & Multiplex Kit from 10x Genomics. We aimed to profile 5,000 cells per library if sufficient cells were retained during dissociation. All libraries were sequenced on Illumina NextSeq until sufficient saturation was reached. After quality control, raw sequencing reads were aligned to the human reference genome GRCh38 and processed to a matrix representing the UMIs per cell barcode per gene using CellRanger (10x Genomics, v3.1). For allograft-derived cells, previously published human single-cell RNA-sequencing data from 7 kidney allograft biopsies were used. The associated raw counts or matrices were downloaded from the Gene Expression Omnibus (GEO, GSE145927, https://www.ncbi.nlm.nih.gov/geo) for ABMR samples (9) and the Kidney Precision Medicine Project (https://atlas.kpmp.org/repository) for No ABMR samples. Filtered gene expression matrices from online data were merged and analyzed using the Seurat R package, version 4 (10). Briefly, cell matrices were filtered with the following parameters: cells with <400 and >10,000 detected genes and >25% mitochondrial transcripts were excluded. After filtering, all objects were integrated using 3,000 features. A full dataset UMAP was generated using Seurat's DimPlot function using the top 17 principal components. Clusters were built using the FindNeighbors and FindClusters functions in Seurat (resolution = 0.6). Cluster identification was performed using the FeaturePlot function by evaluating the expression of specific markers in each cluster as previously described (11, 12). UMPA, dot plots and violin plots were generated using the DimPlot, DotPlot and VlnPlot functions, respectively, in Seurat, with normalized counts in the RNA assay as input data.

### CellChat analysis

CellChat uses a mass action-based model for quantifying the communication probability between a given ligand and its cognate receptor and takes into consideration the proportion of cells in each group across all sequenced cells and expressed co-factors (https://github.com/sqjin/CellChat) (13). Seurat objects encompassing all of the allograft-derived cells or PBMC were used to generate corresponding CellChat objects. Recently reported LILRA1 and LILRB1 ligand-receptor pairs (14) (Table 1) were added to the CellChat database. The aggregated cell-cell communication network was calculated by counting the number of links or summarizing the communication probability. The contribution of each ligand-receptor pair to the overall signaling pathway was computed and the extractEnrichedLR function was used to extract all significant interactions (L-R pairs) and related signaling genes for all available signaling pathways (CellChat_1.1.0 R package).

**Table 1:** LILR-reported ligands interactions.

**Interaction**	**Pathway name**	**Ligand**	**Receptor**	**Evidence**	**Annotation**
HLA-A_LILRB1	LILR	HLA-A	LILRB1	van der Touw et al. (14)	Cell-cell contact
HLA-B_LILRB1	LILR	HLA-B	LILRB1	van der Touw et al. (14)	Cell-cell contact
HLA-C_LILRB1	LILR	HLA-C	LILRB1	van der Touw et al. (14)	Cell-cell contact
HLA-A_LILRA1	LILR	HLA-A	LILRA1	van der Touw et al. (14)	Cell-cell contact
HLA-B_LILRA1	LILR	HLA-B	LILRA1	van der Touw et al. (14)	Cell-cell contact
HLA-C_LILRA1	LILR	HLA-C	LILRA1	van der Touw et al. (14)	Cell-cell contact
CD47_SIRPA	LILR	CD47	SIRPA	van der Touw et al. (14)	Cell-cell contact
S100A8_LILRB1	LILR	S100A8	LILRB1	van der Touw et al. (14)	Cell-cell contact
S100A9_LILRB1	LILR	S100A9	LILRB1	van der Touw et al. (14)	Cell-cell contact
BST2_LILRA4	LILR	BST2	LILRA4	van der Touw et al. (14)	Cell-cell contact

### RNA sequencing

Public data were used for purified monocytes and their differentiated counterparts (GSE146028) (15). Briefly, row count matrices were curated to focus on populations of interest (monocytes, M0, M1, M2, Mreg macrophages). BIOMEX workflow was used to normalize the data and to perform principal component analysis (PCA) and differential expression analysis (16).

### miRNAs analysis

The MIENTURNET (17) web tool was used to interrogate both TargetScan and MiRTarBase databases and to find predicted and experimentally validated miRNA-target interactions. Only interactions with a false discovery rate < 0.25 were considered. The Fantom 5 database (18) was used to determine the level of miRNAs of interest in a curated panel of cell populations.

### Data analysis

We report descriptive statistics using means for continuous variables or numbers. R studio (version 1.3.1073 *Giant Goldenrod*) and GraphPad Prism (version 9, San Diego, CA, United States) were used for statistical analyses and data interpretation. Chord diagrams and heatmaps were obtained using the following packages, circlize (version 0.4.14) and heatmap3 (version 1.1.9), respectively. *P*-values were calculated based on the Student's *t*-test and were corrected with the Bonferroni method. A *P*-value ≤ 0.05 was considered significant.

## Results

### LILRs are mainly expressed by non-classical monocytes in the blood after kidney transplantation

**Figure 1:**
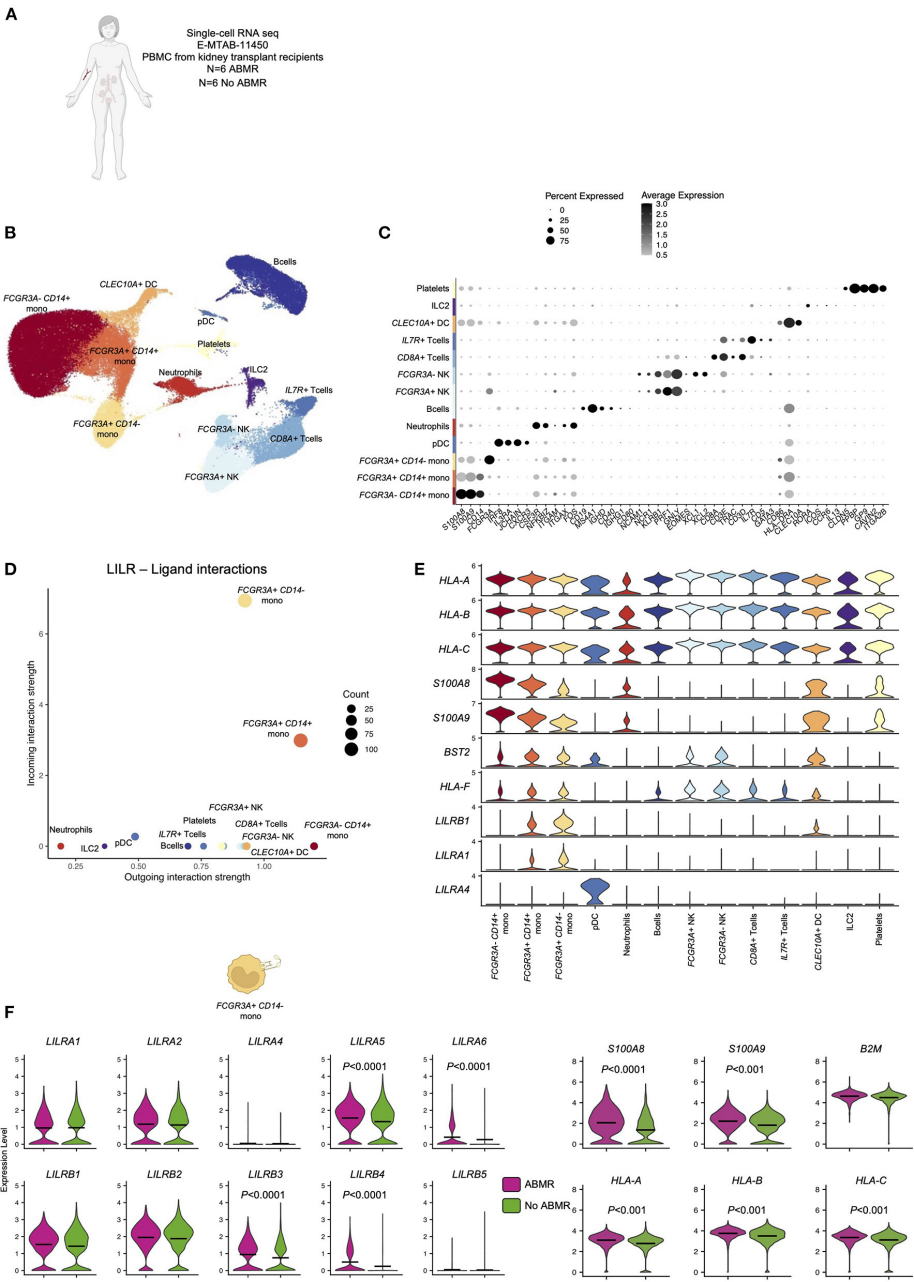
Overview of the single-cell RNA-sequencing analysis on 12 peripheral blood samples with and without antibody-mediated rejection (ABMR) to map LILR expression. **(A)** Briefly, scRNAseq performed on 6 peripheral blood samples from kidney transplant recipients with a concomitant diagnosis of ABMR, and 6 stable patients without ABMR was reanalyzed (E-MTAB-11450). **(B,C)** Unsupervised clustering revealed 13 clusters corresponding to the main myeloid and lymphoid cells and granulocytes/platelets. **(D,E)** CellChat analysis was performed and focused on LILR-ligand. **(D)** The number of incoming and outgoing LILR-ligand interactions is plotted per cell type. **(E)** Violin plots depicting expression of indicated genes in all the cell types. **(F)** We subclustered *FCGR3A*+ *CD14*- monocytes and performed differential expression for ABMR vs. no ABMR for the indicated genes. *P*-values were subjected to Bonferroni correction method.

In order to test the hypothesis that LILRs might contribute to ABMR, we performed a deep immunologic and transcriptional analysis of circulating blood immune cells across a cohort of confirmed ABMR patients (E-MTAB-11450, *N* = 12, 6 patients with ABMR and 6 patients without ABMR, Figure 1A) (8). All of the immune cell clusters were identified and annotated using specific markers (Figures 1B,C), as previously described (8). Four clusters were composed of myeloid cells, with *FCGR3A- CD14*+ classical monocytes, *FCGR3A*+ *CD14*+ intermediate monocytes, *FCGR3A*+ *CD14*- non-classical monocytes and *CLEC10A*+ dendritic cells. Six lymphoid clusters were also identified: a large *CD19*+B cell cluster, but also *CD3D*+*IL7R*+ and *CD3D*+*CD8A*+ T cells; *NCR1*+*FCGR3A-* and *NCR1*+*FCGR3A*+ NK cells, *IL3RA*+ pDC and *RORA*+ ILC2. Two clusters corresponding to *PPBP*+ platelets and *CSF3R*+ neutrophils were also present. Second, we determined which cell cluster was acting dominantly through LILR-ligand interactions in the blood of kidney transplant recipients (KTR). We found that *FCGR3A*+ *CD14*- monocytes were the main cells expressing LILRs (Figure 1D). More specifically, they highly express *LILRA1* and *LILRB1* but also their reported ligands, *HLA-A, B, C, F* and *S100A8, S100A9* (7, 14, 19), suggesting that they can exert both *cis* and *trans* signaling of LILRs (20). These monocytes also express *BST2*, which codes for the ligand of *LILRA4*, mainly expressed in pDCs (Figure 1E). These results indicate that *FCGR3A*+ *CD14*- non-classical monocytes are the main circulating blood immune cells able to respond to allogeneic class I HLA. Comparing the expression of members of the LILRA family and LILRB family in this cell population, we noticed a significant increase in *LILRA5, LILRA6, LILRB3*, and *LILRB4*, suggesting that the tonic regulation provided by the LILRB family might be perturbed in these cells in ABMR (Figure 1F).

### LILR expression is strictly restricted to myeloid cells within the allograft

**Figure 2:**
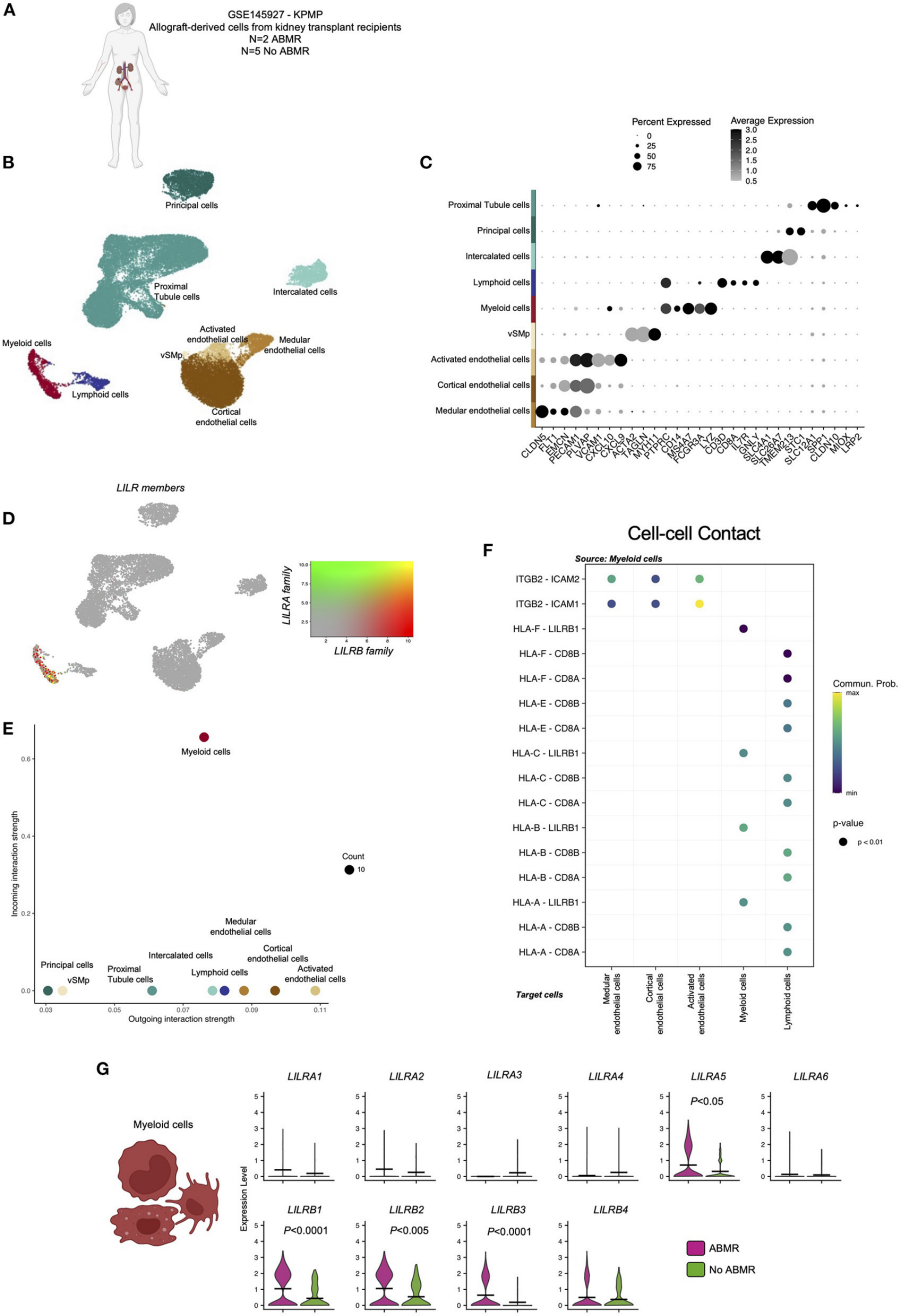
Overview of the single-cell RNA-sequencing analysis on 7 kidney biopsy samples with and without antibody-mediated rejection (ABMR) to map LILR expression. **(A)** Briefly, scRNAseq performed on 2 biopsies from kidney transplant recipients with a concomitant diagnosis of ABMR, and 5 stable patients without ABMR was reanalyzed (GSE145927 and KPMP). **(B,C)** Unsupervised clustering revealed 9 clusters corresponding to the main endothelial cells (EC), myeloid and lymphoid cells but also epithelial renal cells. **(D)** LILR members were plotted on the UMAP. **(E,F)** CellChat analysis was performed and focused on Cell-cell contact signaling. **(E)** The number of incoming and outgoing LILR-ligand interactions is plotted per cell type. **(G)** We subclustered myeloid cells and performed differential expression for ABMR vs. no ABMR for the indicated genes. *P*-values were subjected to Bonferroni correction method.

The infiltration of recipient-derived leucocytes in the allograft in ABMR is well characterized. Two ABMR-specific acute histological lesions are scored based on this cell infiltrate in the microvascular compartment of the allograft: glomerulitis and peritubular capillaritis. We recently showed that monocytes could represent 20–80% of the immune infiltrate in ABMR (21). In order to map the expression of the LILRs within the allograft, we took advantage of publicly available sc-RNAseq datasets (*N* = 7, 2 patients with ABMR and 5 patients without ABMR, Figure 2A) as previously described (6, 11, 12). Regarding tissue-specific cells, we identified three main epithelial renal cell clusters (*LRP2*+ proximal tubule cells, *TMEM213*+ principal cells, *SLC26A7*+ intercalated cells), three endothelial cells (EC) clusters (*CXCL9*+ activated EC, *PLVAP*+ cortical EC, *CLDN5*+ medular EC), a cluster corresponding to vascular and smooth muscle cells (*MYH11*+ vSMp). Immune cells were encompassed in two clusters: *CD3D*+ lymphoid cells and *MS4A7*+ myeloid cells (Figures 2B,C). After plotting the expression of all LILR members in the UMAP, we noticed that LILR expression is restricted to myeloid cells in the allograft (Figures 2D,E). We then better characterized all the significant cellular interactions that took place in the allograft between myeloid cells and other cells.

Interestingly, myeloid cells mainly interact with activated EC through the ITGB2-ICAM1 axis (Figure 2F). Regarding LILR-ligand interactions, only class I HLA-LILRB1 was significantly enriched.

When we compared LILR expression according to clinical outcome, we observed a significant increase in *LILRA5, LILRB1, LILRB2*, and *LILRB3* in myeloid cells in ABMR (Figure 2G).

### LILRs are mainly expressed by non-classical monocytes within the kidney allograft

**Figure 3:**
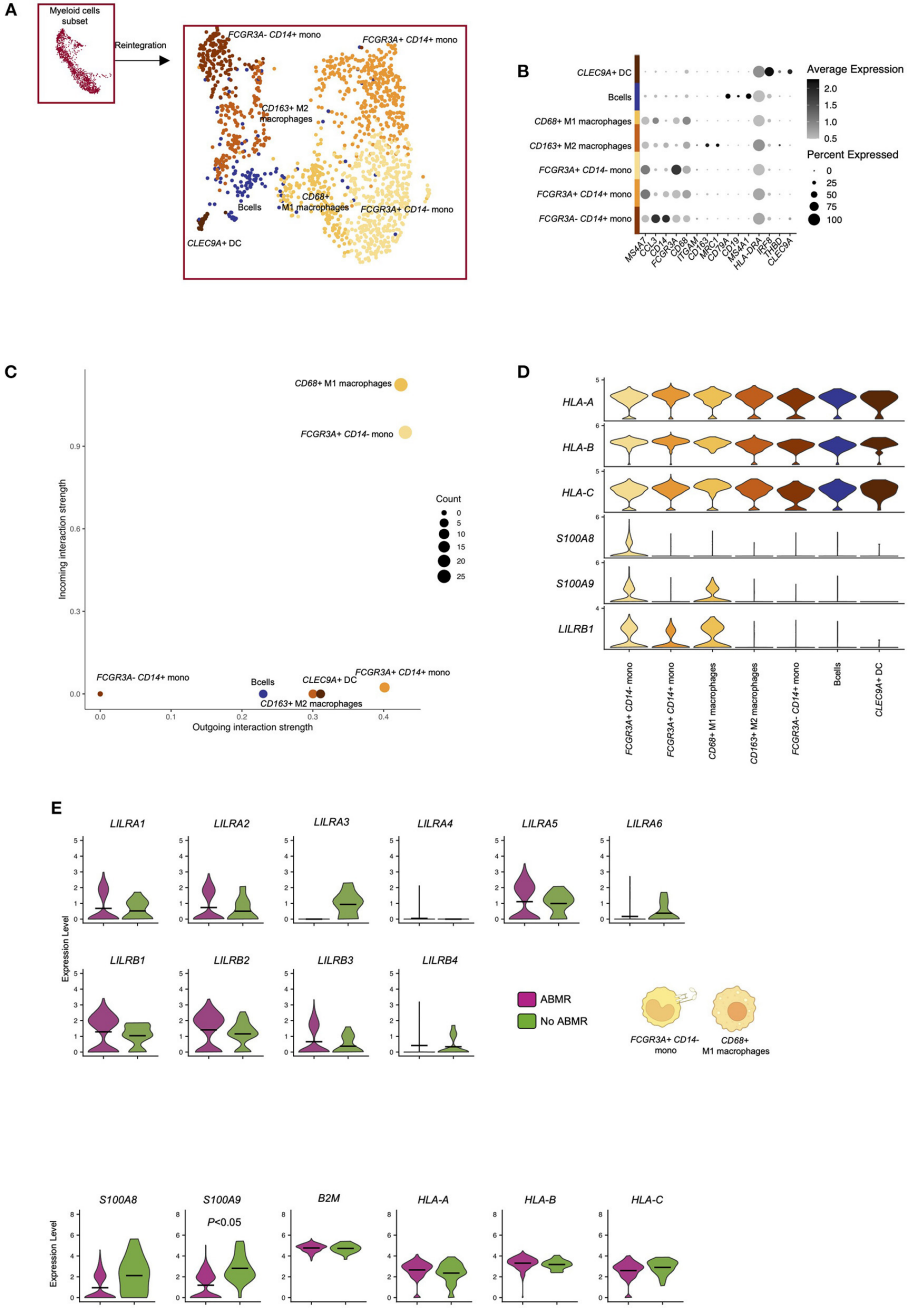
Reintegration of biopsy-derived myeloid cells to map LILR expression at single cell level. **(A,B)** Briefly, myeloid cells were subclustered and reintegrated before subcluster identification and analysis. Unsupervised clustering revealed 7 clusters corresponding to indicated subpopulations. **(C)** CellChat analysis was performed and focused on LILR signaling. The number of incoming and outgoing LILR-ligand interactions is plotted per cell type. **(D)** Violin plots depicting expression of indicated genes in all the cell types. **(E)** We subclustered both non-classical *FCGR3A*+ *CD14*- monocytes and *CD68*+ M1 macrophages and performed differential expression for ABMR vs. no ABMR for the indicated genes. *P*-values were subjected to Bonferroni correction method.

We then reintegrated all the myeloid cells and subclustered them. We thus identified 7 subclusters corresponding to *FCGR3A– CD14*+ classical monocytes, *FCGR3A*+ *CD14*+ intermediate monocytes, *FCGR3A*+ *CD14–* non-classical monocytes, *CLEC9A*+ dendritic cells, *CD68*+ M1 macrophages, *CD163*+ M2 macrophages and a cluster corresponding to a lymphoid population of *CD19*+ B cells, probably due to overlap of antigen-presenting cell function related genes (Figures 3A,B). Interestingly, *FCGR3A– CD14*+ classical monocytes showed very limited LILR-ligand interactions. In contrast, *CD68*+ M1 macrophages and *FCGR3A*+ *CD14–* non-classical monocytes were the two myeloid populations presenting the highest incoming and outgoing LILR-ligand interactions (Figures 3C,D). They both express high levels of *LILRB1* and its ligand *S100A9*. In addition, *FCGR3A*+ *CD14–* non-classical monocytes expressed *S100A8*, another LILRB1 ligand. Comparing LILR expression levels according to the clinical outcome in these two subsets, we confirmed that the increase in *LILRA5, LILRB1, LILRB2*, and *LILRB3* in ABMR is mainly driven by these populations (Figure 3E).

### LILRs expression is strongly impacted during macrophage differentiation

**Figure 4:**
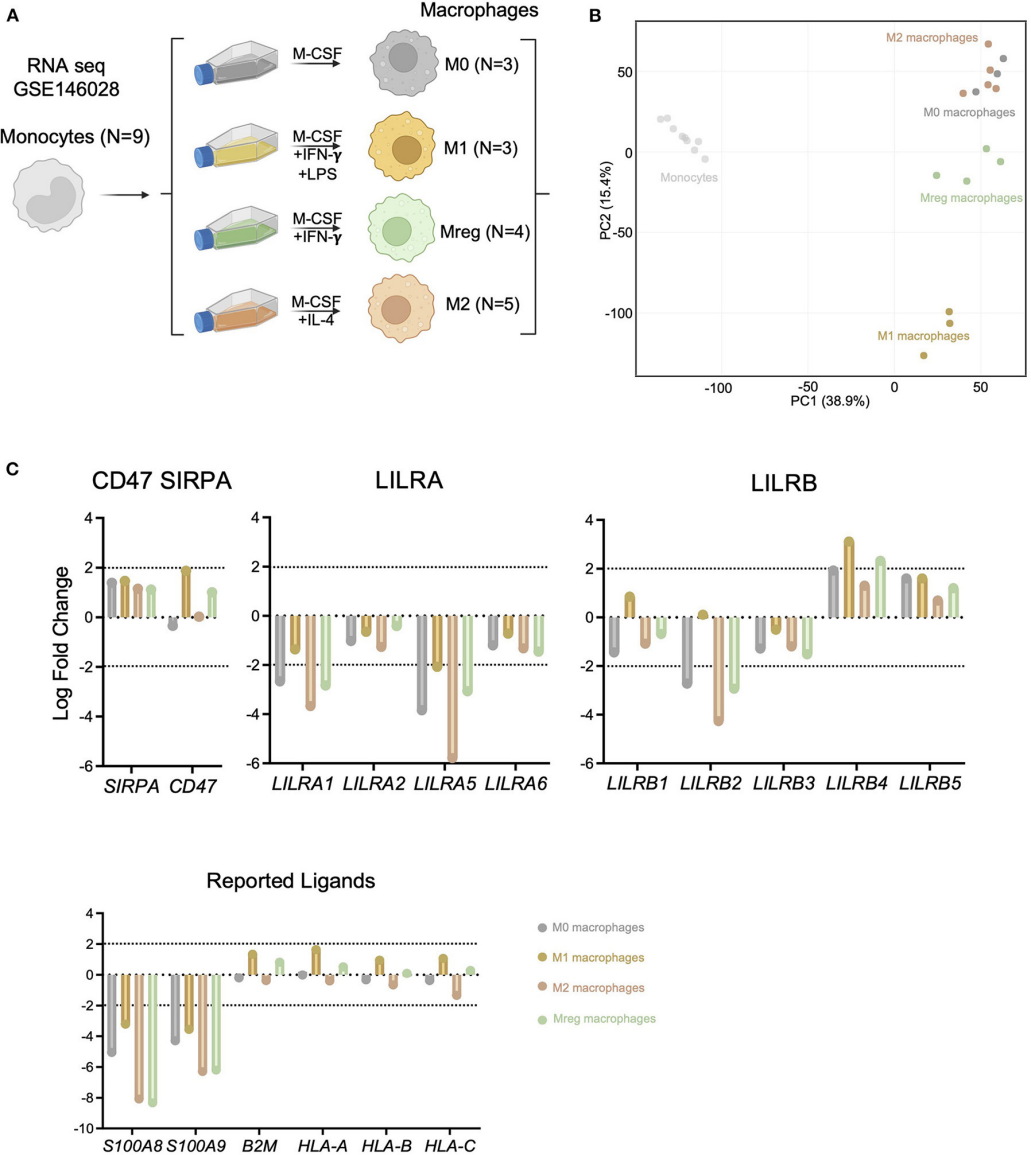
LILRA but also *S100A8* and *S100A9* expression are highly regulated during macrophage differentiation. **(A)** Schematic workflow of transcriptomic comparison. Public data corresponding to undifferentiated monocytes, Mreg, M0, M1 or M2 transcriptomes were analyzed. **(B)** Principal Component Analysis illustrating macrophage differentiation impact at transcriptional level. **(C)** Expression of the LILRA and LILRB families as well as *CD47* and *SIRPA* and reported ligands in the indicated differentiated cells (Log Fold change vs. Monocytes). Log Fold change > |2| were depicted by dotted lines.

Given that circulating recipient-derived monocytes infiltrate the allograft and differentiate into macrophages (22, 23), we assessed the differential expression of LILRs during macrophage differentiation. In this aim, we reanalyzed public RNA sequencing data from purified monocytes and their differentiated counterparts (15), as depicted in Figure 4A. These datasets included RNA extracted from monocytes (*N* = 9 donors) or monocyte-derived macrophages from the same donors differentiated with M-CSF (M0, *N* = 3), M-CSF and LPS and IFN-γ (M1, *N* = 2), M-CSF and IFN-γ (Mreg, *N* = 4) or M-CSF and IL-4 (M2, *N* = 5) according to a clearly-defined protocol (24). Interestingly, macrophage differentiation induced strong transcriptional changes. In line with a previous report, M2 and M0 transcriptomes were quite similar whereas Mreg transcriptomes formed a separate cluster, and M1 transcriptomes were clearly different from other macrophages (Figure 4B). When we focused on the genes responsible for myeloid allorecognition priming, such as *CD47* and *SIRPA* (25), we found just a slight increase in *CD47* after M1 differentiation (Log Fold Change >1.8). Regarding the LILRA family, an overall decrease in LILRA expression was noticed during macrophage differentiation, and more particularly there was a significant decrease (Log Fold Change < –2) in *LILRA1*and *LILRA5* in M0, M2, and Mreg macrophages. In contrast, LILRB members were differentially impacted by macrophage differentiation: *LILRB2* expression was significantly decreased (Log Fold Change < –2) in all macrophages except M1, and *LILRB4* was increased during M1 and Mreg differentiation. For the reported ligands of LILRs, we saw a notable decrease in *S100A8* and *S100A9* coding for LILRB1 ligands during macrophage differentiation regardless of cell polarization. In contrast, the expression levels of *B2M, HLA-A, HLA-B*, and *HLA-C* were not impacted by macrophage differentiation (Figure 4C).

### LILRs ligands are putative targets of miRNAs

**Figure 5:**
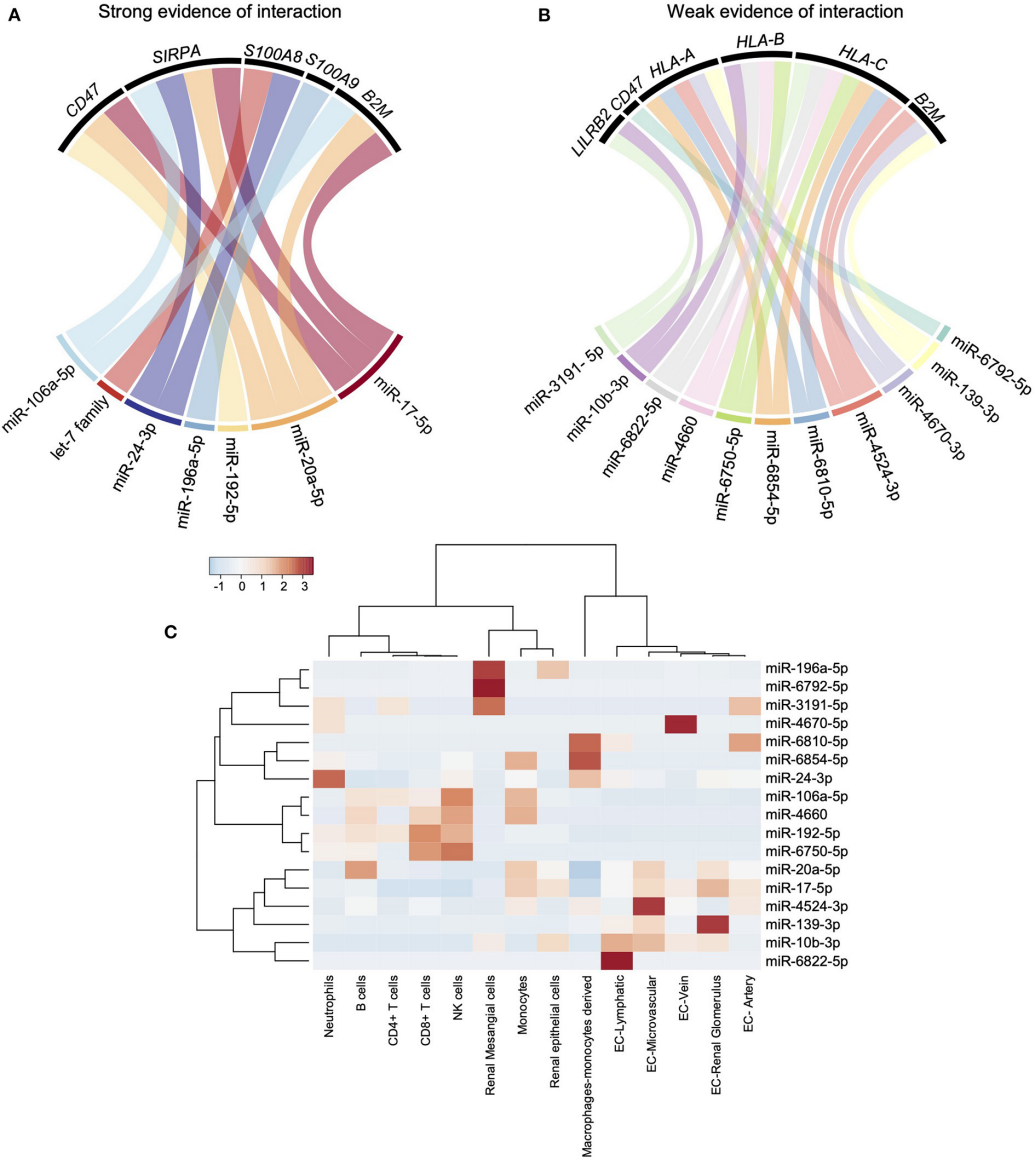
miRNAs strongly regulate LILR reported ligands but not LILRs. **(A,B)** MiRTarBase and TargetScan databases were interrogated using MIENTURNET web tool. Strong evidence of interaction (predicted or experimentally validated with robust methods) and weak evidence of interaction (experimentally validated with immunoprecipitation) were depicted using chord diagrams. **(C)** The Fantom 5 database was interrogated to characterize the main cellular sources of the miRNAs of interest and a heatmap was built.

In solid-organ transplantation, small non-coding microRNAs (miRNAs) have emerged as key players in the regulation of allograft cells function in response to injury (11, 26). We interrogated both MiRTarBase and TargetScan using the MIENTURNET web tool to assess the potential miRNAs targeting LILRs or their reported ligands (17). TargetScan predicts the biological targets of miRNAs by searching for the presence of conserved sites that match the seed region (i.e., the region comprising nucleotides 2–7 at the 5′-end of the mature miRNA sequence) of each miRNA (27). In addition, miRTarBase collects miRNA-target interactions that have been validated by reporter assay, western blot, microarrays, and next-generation sequencing experiments (28). Unexpectedly, no strong evidence provided by robust experimental methods (e.g., Luciferase assay, Western) or prediction was reported for any LILRs. In contrast, some of their ligands, such as *CD47, SIRPA, S100A8, S100A9*, and *B2M*, were suggested as targets of one or more miRNAs (Figure 5A). Considering the weak evidence regarding interaction (resulting from crosslinking and immunoprecipitation experiments), *LILRB2* was the only LILR regulated by miR-3191-5p and miR-10b-3p. In addition, *HLA-A,-B* and*-C* were both regulated by two or more miRNAs such as miR-6854-5p or miR-6810-5p (Figure 5B). We interrogated the Fantom 5 database (18) to explore the cell origins of all these miRNAs. Interestingly, monocytes expressed high levels of miR-6854-5p, miR-106a-5p, miR-4660, miR-20a-5p, and miR-17-5p. In contrast, there was a profound decrease in miR-106a-5p, miR-4660, miR20a-5p, and miR-17-5p in differentiated macrophages concomitantly with an increase in miR-6854-5p and miR-6810-5p (Figure 5C). Altogether, these results highlight new candidates that may potentially orchestrate human myeloid allorecognition mechanisms.

## Discussion

The pathogenic involvement of certain myeloid populations in kidney transplant rejection has been known for decades (22, 23), with primary involvement of monocytes and macrophages in allorecognition through antibody-dependent cell cytotoxicity, after anti HLA-DSA binding in a context of ABMR (29). However, experimental murine models have shed new light on the fact that myeloid cells can respond to allogeneic tissue through an adaptive immunity-independent pathway (30). In mice, myeloid allorecognition is primed through donor polymorphic SIRP-α sensing by recipient CD47 (25). This binding enhances PIRA expression at the surface of myeloid cells, making them able to bind allogeneic class I MHC, which contributes to myeloid cell activation and leads to graft rejection (4). In human solid organ transplantation, the role of LILRs, the PIR orthologs, has not been deciphered yet. More precisely, unusual recognition of individual HLA class I alleles by LILRs may alter the overall balance between activating and inhibitory signals within myeloid cells (31) and subsequently contribute to the development of allograft rejection. In this study, we mapped the expression of the different LILRs and of their reported ligands using single cell RNAseq and RNA seq data. We observed that, in peripheral blood, LILRs are mainly expressed by non-classical monocytes after kidney transplantation. In this cell population, we noticed an increased expression of *LILRA5, LILRA6, LILRB3*, and *LILRB4* in patients with ABMR. In mice, members of the PIRA family were the main drivers of allorecognition (4). We can speculate that the overexpression of *LILRA5* and *LILRA6* in ABMR could result from anterior CD47/SIRP-α sensing, but this hypothesis needs to be validated in further experiments. We also observed an increase in *S100A8, S100A9* and class I HLA genes, suggesting that cis-activation through LILRB1 and LILRB2 may be elevated in circulating non-classical monocytes and regulate their activation in ABMR.

Within the allograft, only myeloid cell populations expressed LILRs, and we found that *LILRA5, LILRB1, LILRB2*, and *LILRB3* were increased in patients with ABMR. This profile was confirmed in non-classical monocytes infiltrating the graft and in differentiated CD68+ M1 macrophages. Interestingly, agonizing LILRB3 in humanized mice was recently reported as tolerogenic signaling that enabled the efficient engraftment of allogeneic cells (32). Therefore, confirming the increase of LILRB3 in the context of ABMR and investigating its potential pathophysiological role appears to be a promising avenue of investigation. In addition, in contrast to what was observed in the blood compartment, *S100A9* expression was decreased within the allograft in patients with ABMR. These results echo our findings based on *in vitro* data showing that *S100A8* and *S100A9* are profoundly repressed during macrophage differentiation. We can thus speculate that cis-activation by the LILRB1/S100A8-9 axis at the surface of monocyte cells is repressed during macrophage differentiation and that the LILRB1/S100A8-9 balance is dysregulated in ABMR.

Finally, we assessed the expression of potential modulators of LILR expression, such as miRNAs. Indeed, miRNAs have been found to play critical roles in many biological processes by controlling gene expression at the post-transcriptional level. They appear to fine-tune the immune response by targeting key regulatory molecules especially in monocytes (33). Surprisingly, we found no prediction of LILR regulation by miRNA. Only weak evidence based on immunoprecipitation experiments suggested that *LILRB2* is regulated by miR-10b-3p and miR-3191-5p, but these miRNAs were barely expressed by myeloid cells. Regarding LILR reported ligands and *CD47/SIRPA*, we found that the let-7 family, miR24-3p, and miR-196a-5p can regulate *S100A8* and *S100A9* activity through posttranscriptional modulation. Interestingly, miR-17-5p and miR-20a-5p were both experimentally confirmed as modulators of *CD47, SIRPA*, and *B2M*. These two miRNAs were elevated in monocytes but strongly decreased in macrophages, suggesting that *CD47, SIRPA*, and *B2M* were no longer repressed in macrophages.

Our study presents several limitations. We reanalyzed public RNAseq dataset to decipher the impact of monocyte-to-macrophage differentiation in LILR expression but these cells were derived from healthy volunteers and we cannot exclude that maintenance immunosuppression influences these findings. Moreover, single cell RNAseq dataset were used to map LILR transcription in blood and kidney biopsies, but further experimental validation is required for the associated proteins. Notably, the CellChat analyses based on transcripts expression only predict cell communications and these communications and cell-cell contact should be validated by other methods such as immunostaining. In addition, we highlighted non-classical CD16+ CD14- monocytes as the cell population of interest in kidney transplantation in term of LILRs expression in both blood and allograft, but *in vitro* differentiation analysis and miRNA analyses were performed in classical CD14+ monocytes. Non-classical CD16+ monocyte differentiation is still ill-defined, but further investigation could provide better insight into the workings of the LILR pathways. Of note, our findings indicate that non-classical *FCGR3A*+ monocytes and CD68+ M1 macrophages were mainly involved in LILR biology in kidney transplantation context. A recent report showed that *FCGR3A* and *CD68* expression were clearly attributed to recipient-derived cells but we cannot exclude a potential role of donor-derived resident macrophages in the LILR interactions. Altogether our results emphasize non-classical monocytes and CD68+ M1 macrophages as key players in LILRs-ligand interaction in kidney transplantation and pave the way to future investigations of their potential role in solid organ rejection.

## Data availability statement

Single-cell RNA sequencing data from peripheral blood samples have been deposited at the ArrayExpress database (http://www.ebi.ac.uk/arrayexpress) under the series accession number E-MTAB-11450.

## Ethics statement

The studies involving human participants were reviewed and approved by the CNIL (DR 2025-087 N°914184, 15/02/2015) and the French Ministry of Higher Education and Research (file 13.334-cohort DIVAT RC12_0452, www.divat.fr). Samples were stored in the local Biological Resource Center (CRB) of Nantes, France. The CENTAURE biocollection is declared since 08/13/2008 to the Ministry of Research (N°PFS08-017). The patients provided written informed consent to participate in this study.

## Author contributions

BL and CT designed research studies, analyzed data, and wrote the manuscript. CG, FC, RD, MG, SB, EV, JC, MN, DA, BB, ML, and J-MR edited the manuscript. All authors contributed to the article and approved the submitted version.

## Funding

BL was supported by the MiMédi project funded by BPI France (Grant No. DOS0060162/00) and the European Union through the European Regional Development Fund of the Région Bourgogne Franche-Comté (Grant No. FC0013440).

## Conflict of interest

The authors declare that the research was conducted in the absence of any commercial or financial relationships that could be construed as a potential conflict of interest.

## Publisher's note

All claims expressed in this article are solely those of the authors and do not necessarily represent those of their affiliated organizations, or those of the publisher, the editors and the reviewers. Any product that may be evaluated in this article, or claim that may be made by its manufacturer, is not guaranteed or endorsed by the publisher.
